# Thyroid-Associated Orbitopathy and Biomarkers: Where We Are and What We Can Hope for the Future

**DOI:** 10.1155/2018/7010196

**Published:** 2018-03-15

**Authors:** Natacha Turck, Simone Eperon, Maria De Los Angeles Gracia, Aurélie Obéric, Mehrad Hamédani

**Affiliations:** ^1^Optics Group, Department of Human Protein Sciences, University Medical Center, Geneva, Switzerland; ^2^Department of Ophthalmology, Jules-Gonin Eye Hospital, Fondation Asile des Aveugles, University of Lausanne, Lausanne, Switzerland

## Abstract

**Background:**

Thyroid-associated orbitopathy (TAO) is the most common autoimmune disease of the orbit. It occurs more often in patients presenting with hyperthyroidism, characteristic of Graves' disease, but may be associated with hypothyroidism or euthyroidism. The diagnosis of TAO is based on clinical orbital features, radiological criteria, and the potential association with thyroid disease. To date, there is no specific marker of the orbital disease, making the early diagnosis difficult, especially if the orbital involvement precedes the thyroid dysfunction.

**Summary:**

The goal of this review is to present the disease and combine the available data in the literature concerning investigation of TAO biomarkers.

**Conclusions:**

Despite the progress done in the understanding of TAO disease, some important pieces are still missing. Typically, for the future, major efforts have to be done in the discovery of new biomarkers, validation of the suspected candidates on multicenter cohorts with standardized methodologies, and establishment of their clinical performances on the specific clinical application fields in order to improve not only the management of the TAO patients but also the therapeutic options and follow-up.

## 1. Clinical Significance

Around 25–50% of patients with Graves' disease develop TAO without any predictive factor. Moreover, the ocular disorder usually appears after the thyroid disease or simultaneously but may precede it. Identifying new biomarkers of this orbital disease could help to an early diagnosis, especially if the orbital involvement precedes the thyroid dysfunction.

## 2. Introduction

Thyroid-associated orbitopathy (TAO), also known as thyroid eye disease or Graves' ophthalmopathy, is an autoimmune disease affecting the thyroid, orbits, and skin. Despite important progress in understanding the pathophysiological mechanisms leading to the development of this disease in the orbits during the last decade, some important questions are still without any answer. The exact nature of the relationship of TAO with thyroid remains enigmatic: hyperthyroidism can be related to the development of this orbital disease but exceptions exist. In contrast, TAO can occur in hypo- or euthyroid patients. Therefore, the prediction of Graves' evolution to TAO is difficult and limits early treatment. At cellular and molecular levels, the reason why only orbital fibroblasts (and not the other fibroblasts of the body), orbital adipose tissue, and medial and inferior rectus muscles are more often affected during the disease has not been solved yet. Furthermore, the possibility to have unilateral orbital case and the great variety of clinical presentation are not understood. This last point highlights also in some cases the difficulty to properly diagnose TAO disease by cofounding with mimicking diseases such as orbital myositis, amyloidosis, some tumors or metastatic cancer, and IgG4-related diseases [1–12].

In this context, the discovery of new biomarkers that could definitively assist the physician to diagnose TAO disease as early as possible, predict prognosis, and propose early and appropriate treatment will be clinically useful for improving patient management. After a brief recall of the clinical manifestations and the pathophysiology, we review where we are in the potential biomarkers reported in TAO and which vision we can have for the future.

## 3. Review

The natural history of TAO, without any treatment, is described as Rundle's curve [13–15]. Symptoms and signs of the orbital disease worsen rapidly during an initial phase, reach a maximal severity, and decrease to a plateau known as sequelae. The disease appears 2–6 times more frequent in young women, but severe cases occur more frequently in men more than 50 years old [16].

**Figure 1:**
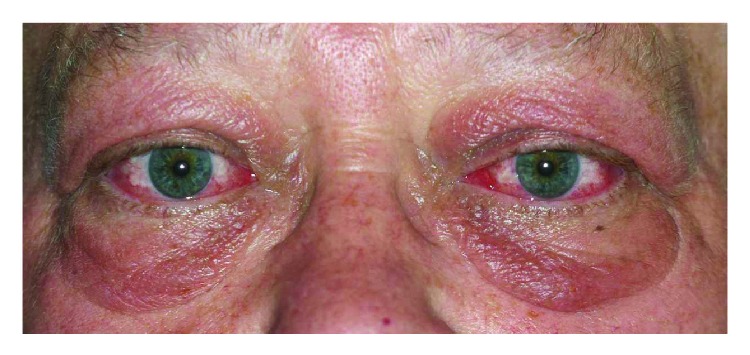
Bilateral inflammatory thyroid-associated orbitopathy with edema and redness of eyes and lids.

**Figure 2:**
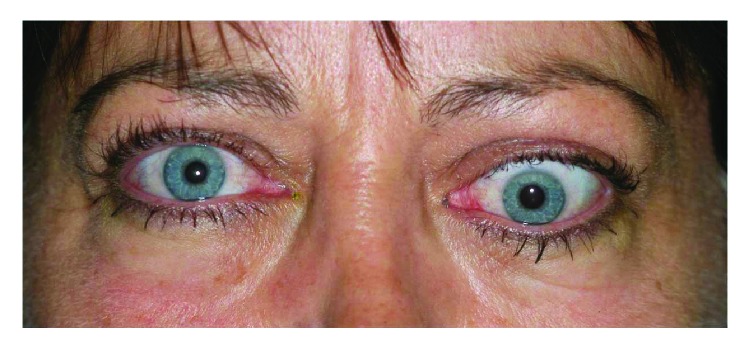
Left unilateral exophthalmos with limitation in upgaze and diplopia (double vision).

The manifestations of the orbital involvement are irritation and redness of the eyes and eyelids, lid tumefaction, double vision, and rarely visual loss. The bilateral complete orbital examination should look for lid retraction, proptosis (exophthalmos), limitation of ocular motility, fat hypertrophy, deficit of visual acuity or color vision, the signs of corneal exposure, and signs of orbital inflammation [17–19] (Figures 1 and 2).

Clinically, the challenge is to recognize the active, inflammatory phase of the orbital disease. In fact, early diagnosis and rapid introduction of the anti-inflammatory treatment, mainly steroids, improve the final outcome and reduce the functional and disfiguring sequelae of the disease [14, 20]. As in some cases the orbital manifestations precede the thyroid dysfunction and its systemic signs [21], it seems essential to have a biomarker dedicated to the early diagnosis of the orbital disease. The detection of thyroid-stimulating hormone-receptor (TSH-Receptor) antibodies (TSH-R-Abs) may confirm the autoimmunity and the diagnosis of TAO. But these antibodies are not present in all cases [19, 22, 23].

So far, we use the clinical activity score (CAS) to determine the indication and the duration of anti-inflammatory treatment [22, 24]. We take in consideration the presence or not of pain, lid and conjunctival edema (chemosis), and lid and conjunctival redness. Nevertheless, as for all the clinical scales, this one presents some limitations: CAS is based on few items, mixing different types of clinical information (inflammation versus vision worsening) and proposing only binary answers, reducing therefore the accuracy of its interpretation. Furthermore, this is a subjective scale depending therefore on the timing of the evaluation, on the willingness and objectivity of the patients regarding their clinical situations, and on the level of expertise of the practitioner performing the evaluation. Other scales exist including NOSPECS [25], VISA [26], and EUGOGO [27] but present also advantages and limitations and are not daily used in our hospital.

In some difficult cases, the magnetic resonance imaging (MRI) could help to find out the presence of an inflammatory process. Definitely, having molecules that could efficiently complement clinical scores and observation could allow a more precise and rapid diagnosis and also limit the economic burden to useless access to imaging.

All the patients with irritation, lid retraction, and proptosis should benefit of a local lubricant treatment (eye drops and ointment). In the presence of orbital inflammation, some treatments such as selenium and steroids are indicated, according to the severity [22]. The goal is to stop the inflammatory process and to improve the final outcome. In case of resistance or contraindication, low-dose external radiotherapy is suggested. The immunomodulatory treatments such as tocilizumab (interleukin- (IL-) 6 receptor antagonist), teprotumumab (insulin-like growth factor-1 (IGF-1) receptor antagonist), or rituximab (anti-CD20) [28] seem to give promising results in resistant cases [18, 29–31].

Rehabilitative surgery should be performed in patients with inactive TAO since at least six months. The main steps are orbital decompression for the reduction of proptosis, squint surgery for the treatment of muscular fibrosis and diplopia, lid lengthening, and blepharoplasty for lid retraction and fat hypertrophy.

The finding of specific biomarkers of TAO may serve as a predictive factor of development of TAO among patients with Graves' disease and may also give some information on the severity of TAO.

The pathophysiology of TAO is poorly understood. Some classical risk factors including genetic predisposition, environmental factors, infection, and stress have been reported, but their real impact on TAO initiation remains debated [32].

Nevertheless, some pieces of the puzzle begin to come together in the literature. B cells, T cells, and orbital fibroblasts have been shown to be the key players of the pathological event. At the origin, T cells are responsible for the initiation of the disease [19]. Indeed, T helper cells become activated when they recognize TSH-R peptides on antigen-presenting cells. Upon interactions with such T cells, B cells secrete anti-TSH-R antibodies. These antibodies lead to stimulation of both thyroid follicular cells, which produce a great quantity of thyroid hormones, and orbital fibroblasts, which proliferate and induce orbital changes.

Beside TSH-R, IGF-1 receptor (IGF-1R) was also identified as a potential targeted antigen [32–37], and it seems that the interactions between TSH-R and IGF-1R are more important than individual molecules' effect [38]. Furthermore, patients can have either one or both types of autoantibodies, and alternative production of other types of autoantibodies is not excluded. Indeed, recent studies suggested that autoantibodies against carbonic anhydrase 1 and alcohol dehydrogenase 1B had higher prevalence in orbital fat in TAO compared to those in controls [39].

Tripartite relationships between orbital fibroblasts, B cells, and T cells initiate cascades of immune and chemical reactions [40, 41] resulting in pathological situations: inflammation of the connective tissues, fibrosis, and adipogenesis [32, 33].

These phenomena cause fundamental and dramatic ocular tissue remodeling. The increased volume of extraorbital muscles, induced by intensive hyaluronic acid (HA) production [42] and expansive growth of adipose tissue via activation of peroxisome proliferator-activated receptor gamma (PPAR-*γ*) [43], leads consequently to the typical eye's protrusion, characteristic of TAO patients. In addition, the compression of orbital tissue causes a compression of vascular structures leading to the reduction of blood flow and subsequent localized hypoxia [44]. In this context, proangiogenic factors seem to be stimulated in order to restore appropriate circulation through the formation of new vessels.

### 3.1. Biomarkers

In this context, as previously mentioned, no accurate molecular tool to date allows establishing a rapid, early, and robust diagnosis of TAO or predicting the outcome or the efficiency of drug therapy. Nevertheless, the availability of these kinds of tools, objectively measurable and easily interpretable, could greatly enhance the management of TAO patients, especially those with normal thyroid function. However, over the years and regarding the increased number of publications in the biomarker field, only relatively few studies have been focused on the discovery of new biomarkers in TAO disease.

The term “biomarker” was officially and accurately defined 15 years ago as a single indicator “that objectively measures and evaluates normal or pathogenic biological processes” [45]. Consequently, a biomarker is not restricted to being a protein but may be any type of specific molecular signature such as a gene, mRNA, or a metabolite. Specific clinical features such as demographic and physiological parameters (age, gender, smoking status, or goiter size), imaging (thyroid volume with ultrasonography or IRM), or clinical scores (CAS; vision, inflammation, and appearance (VISA)) can also be considered as objective biomarkers. However, only the molecular biomarkers will be considered here.

In order to be efficient, biomarker discovery in general but also in TAO context should carefully consider the best source of samples in relation to both the clinical question and the methods of investigations. To be applicable on a large scale, a good source of biomarkers should take into account the feasibility of sample collection and its relevance. Extremely invasive sample collection (e.g., biopsy of orbital fat or extraorbital muscles), even if it is highly specific due to the close relationship with the location of a disease, must not be taken for granted because of (i) the related discomfort and risks of secondary complications for the patient; (ii) the restricted access for clinical diagnosis, and (iii) the great difficulty in collecting such samples from healthy control subjects.

### 3.2. Biomarkers—Hormones and Antibodies—in the Blood of TAO Patients

In TAO disease, traditional biological fluids including the blood and urine have been investigated. The majority of the studies rather reported principal actors of the TAO disease as potential biomarkers than discovered new candidates. Considering the dysfunction of the thyroid gland associated to TAO, the traditional circulating thyroid hormones (TSH, triiodothyronine also known as T3, and thyroxine, called T4) used for diagnosing thyroid dysfunction or the antibodies against TSH-R (TSH-R-Abs) [22, 46] or thyroid peroxidase- (TPO-) Abs [47, 48] would be naively expected to be highly studied and give an interesting insight on the clinical status of the TAO patients.

Thus, whatever the generation of assays used, TSH-R levels were shown to be associated to activity and severity of TAO [46, 49–51]. The new-generation tests allowed to reach up to 97% sensitivity and almost 90% specificity [51]. However to date, some limitations persist for a clinical use of TSH-R in the management of TAO. The heterogeneous pattern of thyroid dysfunction in TAO patients—hyperthyroidism, euthyroidism (6 to 21% depending on the studies [52–54]), or hypothyroidism—and the fact that various other diseases [55] may disturb thyroid hormones greatly limit their clinical relevance in TAO diagnosis. Indeed, a potential interference of treatment with the TSH-R level has been suspected [56–58] and could disturb their performances in TAO prediction.

In conclusion, conflicting data related to different types of generation assays and various experimental designs do not allow to definitively evaluate the clinical performance of TSH-R-Ab on TAO patients, and the conditions of its routine use remain to be clarified. In the same context, the association of TPO-Ab and TAO is still questionable as different studies reported various results [47, 59–61].

### 3.3. Biomarkers—Cytokines and Others—in the Blood of TAO Patients

As the pathology is driven by an acute inflammatory event, the proinflammatory cytokines/chemokines including IL-1*β* [62], IL-6 [63], IL-10 [63], IL-8 [64], C-C chemokine ligand 20 (CCL20) [65], and IL-17 [66] have been studied. The reported data reveal an elevation of their level in the blood of TAO patients compared to that of control patients that could highlight a potential interest of these molecules as diagnosis markers. Furthermore, their levels seem even able to determine the stage of the disease: an active phase is characterized by a higher level of IL-1*β*, IL-6 [62], and IL-17 [66] compared to inactive phase. The data suggested also that the blood levels of some cytokines could reflect the response to treatment: patients presenting refractory TAO have higher level of IL-4, IL-6, and IL-10 than patients in remission [63]. Furthermore, patients present modified blood level of IL-16 (increase) and IL-8 (decrease) after steroid treatment compared to the previous state [64, 67]. Moreover, a possible association of serum IL-10 polymorphism with incidence of TAO has been reported [68].

Based on only two unique studies, controversial data exist on interferon-*γ* (IFN-*γ*) and its potential disturbance in the blood of TAO patients [62, 69]. The cytokines involved as mediators of B cells and/or T cells have also been largely investigated due to the key roles of these cells in the initiation and the course of the TAO disease. Interleukin-2 [68], IL-16 [67], and IL-33 [69] have been shown to be highly elevated in the blood of TAO patients compared to those of the controls. Serum IL-33 levels were positively correlated with T3 and T4 however negatively correlated with TSH [69]. A polymorphism of IL-2 is suggested to be associated with the disease [68].

Due to their mitogenic and angiogenic properties, the potential value of growth factors has also been investigated. Serum hepatocyte growth factor (HGF) increases in TAO patients compared to that in control subjects and is sensitive to efficient glucocorticoid treatment. Its level decreases in response to drug administration [64]. Adhesion molecules belong to another class of molecules investigated as potential TAO markers. They play a role in cell/cell or cell/extracellular matrix interaction, activation, and migration. Intercellular adhesion molecule-1 (ICAM-1) and soluble vascular cell adhesion molecule-1 (sVCAM-1) have been found elevated in the blood of TAO patients as compared to those in control patients, but their levels seem also to be influenced by the treatment [70].

Selenium is a metabolite implicated in thyroid hormone synthesis and metabolism [71], both actions having high importance in TAO development [72]. Besides, high amounts of selenium are found in the thyroid gland. In an Australian population in 2014, TAO patients showed lower levels of selenium in serum than patients suffering from Graves' disease without eye involvement. In addition, selenium levels decrease with TAO increasing severity. The authors conclude that the lack of selenium might be an independent risk factor for TAO [72].

The potential interest of several exotic biomarkers in TAO recently emerged notably because of the use of omics strategies. Among these emerging candidates, none of them has been deeply evaluated to date, but several can be mentioned for their biological functions that could be directly related to TAO disease. This is the case of osteopontin [65, 73], a multifunctional protein involved in inflammation, cell recruitment, cell adhesion, and remodeling. It is inversely correlated with TSH level and positively with T3 and T4 [73]. Another protein called cytotoxic T lymphocyte-associated antigen-4 (CTLA-4), a member of the immunoglobulin superfamily, which is found on T cell surface, negatively regulates these cells. So far, many studies have been focused on a polymorphism localized on CTLA-4 gene, as a consequence of its implication in autoimmune diseases [74–76]. Finally, HLA-B8, a MHC class I cell surface receptor, has been observed in association with TAO, but its role remains to be elucidated [77, 78].

### 3.4. Biomarkers in the Urine of TAO Patients

The urine and its components have been little investigated as potential source of biomarkers in the context of TAO. However, three compounds showed a potential promising interest and should be more studied in the future. The cotinine level, the main metabolite of nicotine used as marker of tobacco use, seems to correlate in smoker TAO patients with the level of blood TSH-R-Abs, the activity of the disease, and secondary ocular complication after radioiodine treatment [79, 80]. Glycosaminoglycans (GAGs), the most abundant heteropolysaccharides, display urinary levels 2-3 times higher in patients with the active form compared to those in patients with the inactive form [81]. Finally, 8-hydroxy-2′-deoxyguanosine (8-OHdG) has attracted attention of the scientist's community. This metabolite is used to measure DNA damage in oxidative stress, event that was related with various ocular diseases such as TAO. High levels of 8-OHdG were found in TAO patients' urine compared to those in control patients, and 8-OHdG level was related to CAS [82]. In short, 8-OHdG might be a good biomarker in the future to evaluate the presence of oxidative DNA damage and therefore the oxidative stress generated in TAO patients.

### 3.5. Biomarkers in the Blood and Urine of TAO Patients: Conclusions

In conclusion, these 2 common fluids usually explored for biomarker discovery seem disappointing in TAO. Several explanations could be highlighted: at this stage, only few studies focus on the same molecules and, in the main cases, the candidates are investigated not for their potential role as biomarkers but rather for their central role in the pathological events. This is particularly illustrated by the absence of clinical performances (sensitivity, specificity, and positive and negative predictive values) reported in the publications. Nevertheless, with the democratization of the omics methods, we may speculate that, in a near future, new and probably unexpected biomarkers will be discovered and could offer new clinical and management strategies for TAO.

Moreover, no standardized protocol is reported for the evaluation of a specific target, and different clinical questions are frequently assessed with a unique cohort design decreasing the power of the analyses. Another aspect could be that the modifications of molecular levels occurring in response to this disease may be too subtle to be efficiently measured in these systemic fluids. We assume therefore that fluids or tissues geographically close to the place of the disease (the eyes) will be more valuable.

### 3.6. Biomarkers in the Orbital Fat of TAO Patients

Exploring the orbital fat content in TAO patients is, to our point of view, highly relevant since the disease directly affects this tissue. In the orbital fat, the IL-1*β* and IL-6 levels seem to be associated with the smoker status of TAO patients [83]. Besides, a transcriptomics study performed on orbital fat reports a clear upregulation of IFN-*γ* in TAO patients [84]. Transforming growth factor-*β* (TGF-*β*) and fibroblast growth factor (FGF) are elevated in the orbital fat of TAO patients, and levels of these factors are correlated with the severity of the disease. In the family of growth factors, platelet-derived growth factor (PDGF) is probably the most promising at this stage with a central role in the TAO pathological events. Indeed, several studies have reported its overexpression in orbital tissues of TAO patients [85–87], independently of the activity grade of TAO. In addition, specific isoforms of PDGF improve the TSH-R expression on orbital fibroblasts, amplifying the autoimmune reaction against TSH-R [85]. Drugs blocking PDGF signalling allow opening new therapeutic options [87, 88]. Finally, in vitro studies have also highlighted the adipogenic function of PDGF, able to induce the transformation of orbital fibroblasts into adipocytes [89]. This mechanism participates in the extension of orbital tissue during TAO course. Adipogenesis is also induced by IL-1*β* through an increase of cyclooxygenase-2 (COX-2). This enzyme, known to modulate inflammation, is anticipated to be a central element of the active phase of TAO disease. Its mRNA and protein levels have been shown to be overexpressed in orbital fibroblasts of TAO patients, [90] and hyaluronic acid (HA) seems involved in its regulation. Nevertheless, the interest of COX-2 is not definitively assessed as other studies revealed no modification of its expression [91]. On the other hand, at the transcript levels, TGF-*β* receptor, IGF-1, and insulin-like growth factor binding protein-6 (IBP-6) appear to be downregulated [84].

### 3.7. Biomarkers in the Tears of TAO Patients

From our point of view, the most promising fluid for TAO in the future will be probably tears. Surprisingly, until now, tears and their clinical relevance have been poorly studied. With the noninvasive, easy, and rapid collection of samples, tear-based approaches open up new routes for diagnostic methods and for understanding of both ocular and systemic diseases. Tears play a key role in the correct function and health of the eye. Tears are necessary for the lubrication of the eye surface that ensures the appropriate optical properties and for the nutrition and protection of the surrounding tissues. Tears are secreted by the lachrymal glands and contain electrolytes, nucleotides, lipids, metabolites, and proteins. But these components can also be released from the surrounding damaged tissues or by passive transport from the blood. The production and composition of tears are therefore a dynamic system that depends on environmental factors, stimulus, infection, or disease. Consequently, the ability to measure any subtle modification targeting one or several biomarkers in tear contents opens promising opportunities for screening not only ocular but also systemic diseases.

The behaviour of the proinflammatory proteins in the blood could be extrapolated to tears. A profile similar to that observed in the blood can be highlighted in the tears with a net increase of IL-1*β*, IL-6, and IL-17 in active compared to inactive TAO patients [62]. Another important actor of inflammation, tumor necrosis factor (TNF)-*α*, has been measured only in tears. Its concentration is higher in inactive and active TAO patients than that in control ones [62]. Moreover, two polymorphisms (−1031T/C and −863C/A) of TNF-*α* gene have been found in samples from a Japanese population with a dramatic increase in patients with Graves' disease suffering from TAO in comparison to those without TAO. In addition, these polymorphisms seem associated to the severity of TAO [92]. Interleukin-7 has also been reported in tears and orbital fat and is suspected to change according to the different phases of the disease [93]. Finally, using proteomic experiments, potential new candidates have been revealed such as proline-rich-protein members (PROL1/PRP4) involved in the modulation of the microflora of the eye and presenting protective function [94, 95] or S100 calcium-binding proteins (S100A8/S100A9) modulating inflammation and cell adhesion [94, 95].

## 4. Conclusions

The story of TAO biomarkers is just starting: efficient biomarkers used in routine for TAO have still to be discovered. Ideally, they will offer a new opportunity for improving early diagnosis, follow-up, and treatment monitoring. Further, it could help to a better understanding of pathophysiology and permit new personalized therapeutic strategies.

Nevertheless, to be a success story, biomarker discovery should carefully consider the best source of samples in relation to the clinical question and the characteristics of the TAO disease. In order to be extended on a larger scale and finally to the whole population, we strongly believe that a good source of biomarkers should take into account sample collection feasibility. Orbital fat or muscles, even if highly specific, will not be easy to obtain and their collection is an invasive method. They can be collected during a surgery of orbital decompression, which is possible only when the inflammation is calming down. It means also that such samples could not be extrapolated for basic diagnosis nor used as preventive tool. In these situations, common biofluids such as the blood seem to be more appropriate for biomarker investigation. However, considering the past, whatever the disease, there has been little success in translating these findings into clinical applications.

More unusual samples including tears have recently emerged as new global source of biomarkers and could be promising and innovative clinical tests in TAO disease in the near future. Because tear sampling is a noninvasive and rapid method, tear-based approaches open promising avenues for diagnostic method and will allow opportunities for deepening understanding of this challenging orbital disease. In addition, as a complex mixture, tears offer the possibility of discovering not only proteins but also RNA, lipid, and metabolite biomarkers that could interestingly complement the traditional clinical tools available for ophthalmologists.
